# The impact of AI on education and careers: What do students think?

**DOI:** 10.3389/frai.2024.1457299

**Published:** 2024-11-14

**Authors:** Sarah R. Thomson, Beverley Ann Pickard-Jones, Stephanie Baines, Pauldy C. J. Otermans

**Affiliations:** ^1^School of Human and Behavioural Sciences, Bangor University, Bangor, United Kingdom; ^2^College of Health, Medicine and Life Sciences, Department of Life Sciences, Division of Psychology, Brunel University of London, London, United Kingdom

**Keywords:** generative artificial intelligence (gen AI), higher education, students' perspectives, AI tools, teaching and learning

## Abstract

**Introduction:**

Providing one-on-one support to large cohorts is challenging, yet emerging AI technologies show promise in bridging the gap between the support students want and what educators can provide. They offer students a way to engage with their course material in a way that feels fluent and instinctive. Whilst educators may have views on the appropriates for AI, the tools themselves, as well as the novel ways in which they can be used, are continually changing.

**Methods:**

The aim of this study was to probe students' familiarity with AI tools, their views on its current uses, their understanding of universities' AI policies, and finally their impressions of its importance, both to their degree and their future careers. We surveyed 453 psychology and sport science students across two institutions in the UK, predominantly those in the first and second year of undergraduate study, and conducted a series of five focus groups to explore the emerging themes of the survey in more detail.

**Results:**

Our results showed a wide range of responses in terms of students' familiarity with the tools and what they believe AI tools could and should not be used for. Most students emphasized the importance of understanding how AI tools function and their potential applications in both their academic studies and future careers. The results indicated a strong desire among students to learn more about AI technologies. Furthermore, there was a significant interest in receiving dedicated support for integrating these tools into their coursework, driven by the belief that such skills will be sought after by future employers. However, most students were not familiar with their university's published AI policies.

**Discussion:**

This research on pedagogical methods supports a broader long-term ambition to better understand and improve our teaching, learning, and student engagement through the adoption of AI and the effective use of technology and suggests a need for a more comprehensive approach to communicating these important guidelines on an on-going basis, especially as the tools and guidelines evolve.

## 1 Introduction

While AI has been with us for some time, it has advanced significantly in recent years (Mhlanga, 2023) and is now increasingly present in both education and business, driving efficiency and innovation (Brynjolfsson et al., 2023; UK Government, 2023a,b; Noy and Zhang, 2023). This widespread adoption suggests a broad application of uses and future potential. Launched in November 2022 by OpenAI, ChatGPT is a form of AI that uses sophisticated natural language processing (NLP) to generate text and, more recently, verbal conversations (Rahman and Watanobe, 2023). ChatGPT saw unprecedented growth in its user base, reaching 100 million active users within 2 months of launch (Reuters, 2023). Early adopters of AI have expressed positive views about the potential of AI to disrupt numerous industries, such as software development (Haque et al., 2022), with only minimal criticism or concern. Furthermore, empirical research has been undertaken to evaluate the potential advantages of integrating Generative AI tools, such as ChatGPT, into education (Wardat et al., 2023; Noy and Zhang, 2023). Wardat et al. (2023) conducted qualitative research and found that students perceived ChatGPT to offer advantages in mathematical learning by delivering abstract concepts more comprehensively, and that it could scaffold understanding similar to an educator.

However, where concerns have been expressed, they relate to educational contexts in particular, such as students using it to write essays and prepare assignments, and plagiarism in general (Steponenaite and Barakat, 2023). Despite these concerns, the potential of AI in education is substantial and covers areas such as personalized learning experiences, enhanced teaching, and new approaches to education for both students and educators (Kasneci et al., 2023; Rahman and Watanobe, 2023). AI is a megatrend (Haluza and Jungwirth, 2023) with the potential to disrupt traditional practices (Rahman and Watanobe, 2023). To date, research has provided insights into the perceptions of both educators and parents into the impact of AI, which has been generally positive, but expressing the need for balanced usage and further education (Otermans et al., 2024b). However, there has been very little research relating to students and their perceptions.

### 1.1 Student perceptions

Student perception of AI is an important part of understanding the potential impact AI could have on their education and future careers; if they do not perceive it to be helpful, they are likely to resist using it (e.g., Plaut, 2023). Research conducted to date has shown students have mixed perceptions of AI. For example, Keles (2021) explored the views of university students and found that negative perceptions of AI were more significant than positive perceptions. In contrast, when Chan and Hu (2023) explored higher education students' perceptions of AI use in their degrees, they found the reverse; that students had a generally positive attitude toward AI in teaching and learning, and the personalized support it can offer. Research by Jeffrey (2020) supports the later findings, suggesting that the general view of AI among students is positive, but that they have concerns about its rapid development and impact on humankind. Finally, research by Chan and Hu (2023) found that students see AI as generally positive, with the potential to help personalize their learning experiences, and help with writing, idea generation and research. However, there continues to be a thread of concerns, in this case relating to accuracy, privacy, ethics and impact on personal development, among other areas.

### 1.2 Importance to careers

Teaching university students transferable skills is crucial in today's rapidly changing job market, where the ability to adapt and apply knowledge across various contexts is highly valued. Research shows that students who develop strong 21^st^-century skills—such as critical thinking, problem-solving, and collaboration—are more confident in their transition from education to employment and feel better prepared to meet labor market demands (Habets et al., 2020; Otermans et al., 2024a). A diverse “diet” of assessment including authentic tasks that require students to apply the knowledge and skills they acquire is vital in producing graduates who are prepared for the demands of the world of work (Tree et al., 2024). The integration of AI tool use into education is becoming increasingly essential as these technologies continue to reshape industries. AI tools enhance students' ability to analyze data, automate tasks, and innovate, which are all critical for maintaining competitiveness in a technology-driven economy. Studies indicate that students who engage with AI tools not only improve their technical proficiency but also gain a deeper understanding of how to apply their skills in real-world scenarios, further boosting their employability (Jackson and Wilton, 2016). Therefore, by incorporating AI tools as one of the transferable skills developed within the curriculum, universities can better prepare graduates to navigate the complexities of modern careers and thrive in a dynamic professional landscape.

### 1.3 Understanding of institutional academic misconduct policies

A comprehensive understanding of university policies relating to academic integrity, particularly plagiarism, is often lacking among students. A study conducted at an Australian university revealed that only half of the surveyed students had read the policy on plagiarism, and many were confused about what behaviors constitute plagiarism (Gullifer and Tyson, 2014). In New Zealand, students also expressed confusion over what should and should not be considered plagiarism (Adam et al., 2017). This issue is not isolated; research at a US university found that the presence of multiple plagiarism policies contributed to students' difficulties in determining which policies applied to them (Merkel, 2022). Furthermore, understanding of plagiarism procedures may vary across disciplines. For instance, students show better comprehension of procedures related to copying and pasting and paraphrasing compared to translation (Olivia-Dumitrina et al., 2019). Encouragingly, higher levels of understanding of academic misconduct policies significantly predict students' adherence to these practices (Nabee et al., 2020). In fact, students have supported clear university-wide policies on appropriate AI use, including specifying appropriate and inappropriate use cases (Johnston et al., 2024). With the rapid evolution of new tools that may accomplish tasks not currently addressed in university policies, there is a clear need to produce comprehensive, clear, and updated guidelines to prevent unethical use of AI and other emerging technologies.

### 1.4 Related work

Our research aims to add to existing research (Balabdaoui et al., 2024; Kasneci et al., 2023; Rahman and Watanobe, 2023; Otermans et al., 2024b) and address a critical gap in understanding of students' perceptions of the impact of AI on their education and future careers. While existing research has explored the role of tools such as ChatGPT in learning (Kasneci et al., 2023), we seek to approach issues from a student perspective, by identifying students' understanding and current use of AI tools and their held beliefs and perspectives about the role of AI in their future careers. Furthermore, many universities have created AI policies or guidance documents for students on how they can/cannot use AI in their studies. Students' awareness of these policies is another element that will be explored in the study (Otermans et al., 2024b).

Specifically, our study aims to address the following questions:

**RQ1:** How do students at all levels of study currently perceive and use AI tools?

**RQ2:** What are students' impressions of the importance of AI to their future careers?

**RQ3:** What awareness do students have of AI policies within their universities?

## 2 Materials and methods

There were two parts to our study, enabling us to explore both quantitative and qualitative insights: an online survey and a series of in-person focus groups. The survey included both quantitative and qualitative components as some questions were explorative and other questions were follow-up questions to the quantitative ratings to get a more in-depth understanding of why the participant chose that particular rating.

### 2.1 Online survey

#### 2.1.1 Participants

Data collection took place from 3^rd^ October 2023 to 15^th^ January 2024 via Microsoft Forms. Participants were recruited via social media, university discussion boards and the university SONA system for research participation. Participants were told the nature of the research project and assured that their information would be confidential. Participants were offered an incentive to take part: All Bangor participants were entered into a prize draw for one of two £25 shopping vouchers. Year 1 and Year 2 Psychology undergraduate students at either Bangor University or Brunel University London were also offered one SONA credit for taking part. A total of 498 respondents were recruited. From the original 498, 45 were removed at the screening stage for withholding consent or providing duplicate responses, leaving 453 student participants. Tables 1, 2 give a breakdown of the demographics of the participants per university. Other demographics information such as gender, age, and ethnicity, were not collected as part of this dataset.

**Table 1:** Number of participants across institutions and disciplines.

	**Brunel University London**	**Bangor University**	**Total**
Psychology	134	296	430
Sports Science/Psychology (Sport, Health and Exercise)	8	15	23
Total	142	311	453

**Table 2:** Participants' level of study.

	**Brunel University London**	**Bangor University**	**Total**
Year 0	0	1	1
Year 1	73	166	239
Year 2	66	134	200
Year 3	2	6	8
Year 4+	1	4	5
Total	142	311	453

#### 2.1.2 Context of the study

It is important to note how the universities communicated the use of AI to their students. Table 3 gives a breakdown of each university's AI policy from a content design and readability perspective. The table shows Flesch-Kincaid Grade Level (the expected US school grade level needed to read the text; the higher the value, the harder it is to read) and Flesch Reading Ease Score values (the complexity of the text based on sentence length and word length; the higher the value the easier it is to read).

**Table 3:** Full AI policies of both Brunel University London and Bangor University from a content design and readability perspective.

**Measure**	**Brunel University London**	**Bangor University**
Flesch-Kincaid Grade Level	11.8	9.6
Flesch Reading Ease Score	43.9	38.8
Reading Level	College	College
Average words per sentence	18.8	7.2
Average syllables per word	1.7	1.9
Sentences	27	325
Words	507	2,346

In Bangor University, AI policies were communicated centrally by the university. Firstly, a 5-page document was placed online and freely accessible. Second, a condensed synopsis was sent via email as part of a “student bulletin,” a newsletter sent to all students within the university. At Brunel University London, communications went out to students through a webpage on “Using artificial intelligence in your studies.” This was communicated through the student and staff newsletter, social media, and emails. This was a very short guidance webpage covering a few things: What is AI, How do I use AI ethically? When is it appropriate to use AI? Subsequently many academics used this in teaching sessions to explore the topic of “using AI in your degree.”

#### 2.1.3 Instruments

The survey included 33 potential questions a participant may be asked (Appendix A), depending on their responses. For example, if a participant answered “no” to having ever used an artificial intelligence tool, they were not then asked to list which tools they had used. The 33 questions included 5 screening questions, 3 administrative questions (relating to incentives and future research opportunities), 4 demographic questions (university attended, level of study, year of study and degree programme), and 21 questions relating to their use and perceptions of AI: 12 quantitative and 9 qualitative. The estimated completion time for the entire survey was 15 min.

#### 2.1.4 In-person focus groups

Five semi-structured focus groups were held between 5th May 2023 and 16th January 2024 at Brunel University London to provide qualitative insights into student perceptions and behaviors, to understand and contextualize the quantitative data mixed methods research combines quantitative and qualitative approaches to provide a more comprehensive understanding of complex social phenomena (Almeida, 2018; Subedi, 2023). This approach overcomes limitations of single-method studies, enhances validity and reliability, and allows for data triangulation (Subedi, 2023). Participants were Psychology undergraduates, recruited via social media and the SONA participant pool. Participants were told the nature of the research project and assured that all data would be anonymized. All participants were Year 1 and Year 2 Psychology or Psychology (Sport, Health and Exercise) undergraduates. All participants were offered 4 SONA credits for taking part. Demographic information (ethnicity, gender, programme of study and year of study) was captured for all participants in advance of the focus groups. The focus groups were scheduled for up to 60 min and conducted in private rooms in the Division of Psychology. The facilitators used a semi-structured approach that loosely adhered to a discussion guide of 8 questions (Appendix B). The audio of each focus group was recorded. There was at least one facilitator present for each session. In some sessions a second facilitator was present to support with note-taking activities.

A total of 34 participants were recruited (Male = 9, 26.5%; Female = 25, 73.5%). Thirty-two (94.1%) studied BSc Psychology, 2 (5.9%) studied BSc Psychology (Sport, Health and Exercise). In terms of level of study, 22 (64.7%) were year 1, 12 (35.3%) were year 2. For self-categorized ethnicity, 12 (35.3%) were Asian, 11 (32.4%) were white, 5 (14.7%) were black, 3 (8.8%) were other, 2 (5.9%) were Arab, and 1 (2.9%) was mixed. Ethnicity categories were determined according to the categories defined by the Office for National Statistics (Office for National Statistics, 2021). These data were to provide the context of our sample, and not used in any further analyses.

#### 2.1.5 Analysis of the study

The data from the 453 participants were analyzed using SPSS (IBM) (for quantitative data). Dichotomous string variables were coded with binary codes and all 5-point Likert-type scales were coded 1–5, with 1 representing the least likely/least sure response, and 5 representing the most likely/most sure response. A thematic analysis was conducted in Taguette (for the qualitative data in the survey).

For the focus groups, recordings were transcribed verbatim, and a thematic analysis was conducted in Taguette, in accordance with the conventions of Braun and Clarke (2006). Figures were made in Microsoft Excel and R.

#### 2.1.6 Ethical approval

Both Bangor University and Brunel University's ethics committees approved this study (Bangor University ethics study number: 2023-17416; Brunel University ethics reference: 44744-LR-Sep/2023- 47201-2). All data were handled according to data protection and GDPR requirements.

## 3 Results

### 3.1 Online survey

#### 3.1.1 How do students at all levels of study currently perceive and use AI tools?

The first part of our survey was intended to poll students on their awareness of AI tools, the tools they used, and the potential use cases they identified. We used four single-choice questions, one Likert-type scale, and five free-text boxes to explore questions in more detail, including the factors that might discourage students from using AI (see Appendix A).

**Figure 1:**
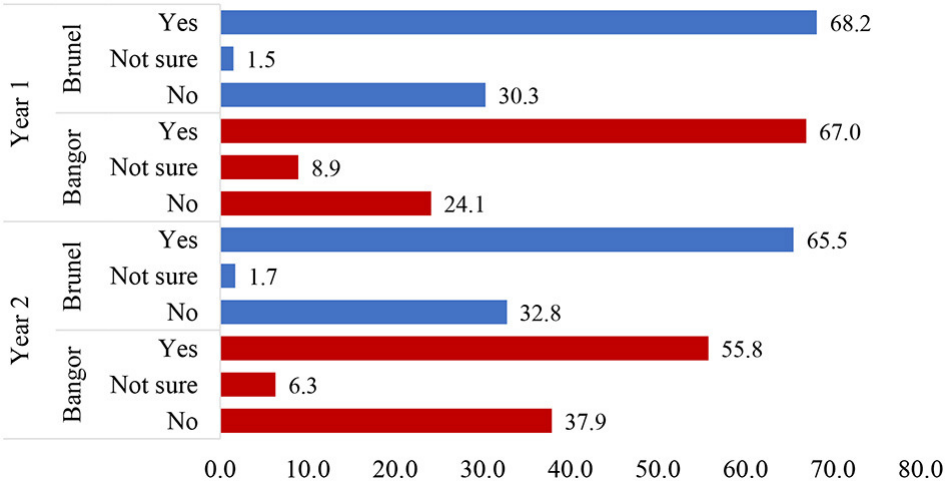
Responses to the question, “Have you ever used an AI tool,” by university and year of study. NB, Only BSc years 1 and 2 from psychology were included due to the low numbers of students in the other categories.

The vast majority (*N* = 430, 94.9%) of the 453 students who responded to our survey had heard of AI tools, with the remaining saying they had either never heard of them (*N* = 12, 2.6%) or were unsure (*N* = 11, 2.4%) whether they had heard of them (see breakdown by university and year of study in Table 4). When we then asked them if they had used AI tools generally, nearly two-thirds had (*N* = 276, 60.9%), while a little under one-third had not (*N* = 144, 31.8%), with 33 (7.3%) not sure (see breakdown by university and year of study in Figure 1).

**Table 4:** Responses to the question “Have you ever heard of AI tools” by university and year of study.

**Year of study**	**University**	**Response**	**Frequency**	**Percent**
Year 1	Bangor	Not sure	1	0.9
		Yes	111	99.1
	Brunel	Not sure	2	3.0
		Yes	64	97.0
Year 2	Bangor	Yes	95	100.0
	Brunel	Yes	58	100.0

NB, Only BSc years 1 and 2 from psychology were included due to the low numbers of students in the other categories.

**Figure 2:**
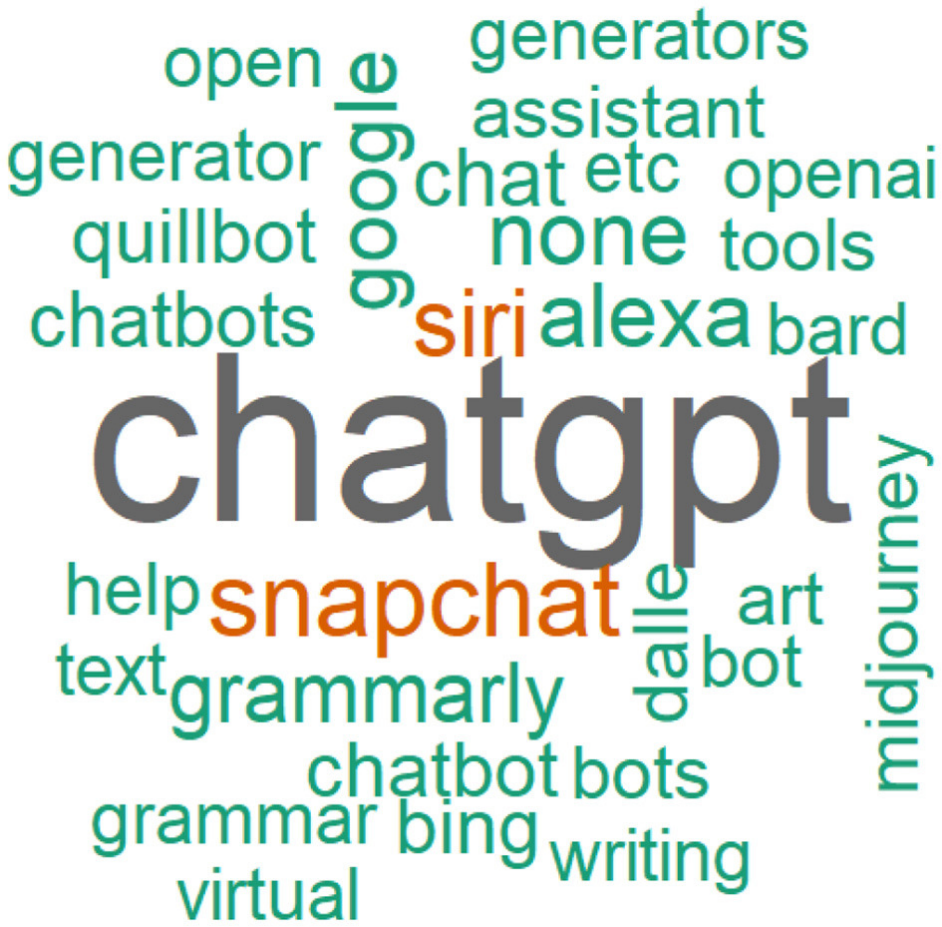
A visualization of the AI tools students used, using their original free-text responses.

Free-text responses were used to query the AI tools used by students. Due to the plethora of tools that could be used and the variations in spellings, we manually categorized the responses as follows: ChatGPT, Snapchat, Grammarly, Google products, Quillbot, Dall-e, Siri/Alexa, Bing, Other, or None. ChatGPT was the most mentioned AI tool they had used (*N* = 182, 40.2%), with other tools mentioned including Snapchat (*N* = 37, 8.2%) and personal assistants such as Siri, Alexa, and Google assistant (*N* = 49, 10.8%). Only 15 (3.3%) participants disclosed using Grammarly, and 36 (7.9%) used a variety of other AI tools e.g., QuillBot and DALL.E 2 (Figure 2).

Of the 276 that responded, more than half of the students (*N* = 147, 53.2%) said they felt very or quite confident in using these AI tools. 91 (33.0%) were neutral/not sure. Thirty-eight (13.8%) were not very confident or not confident at all.

#### 3.1.2 How and why students are using AI

**Figure 3:**
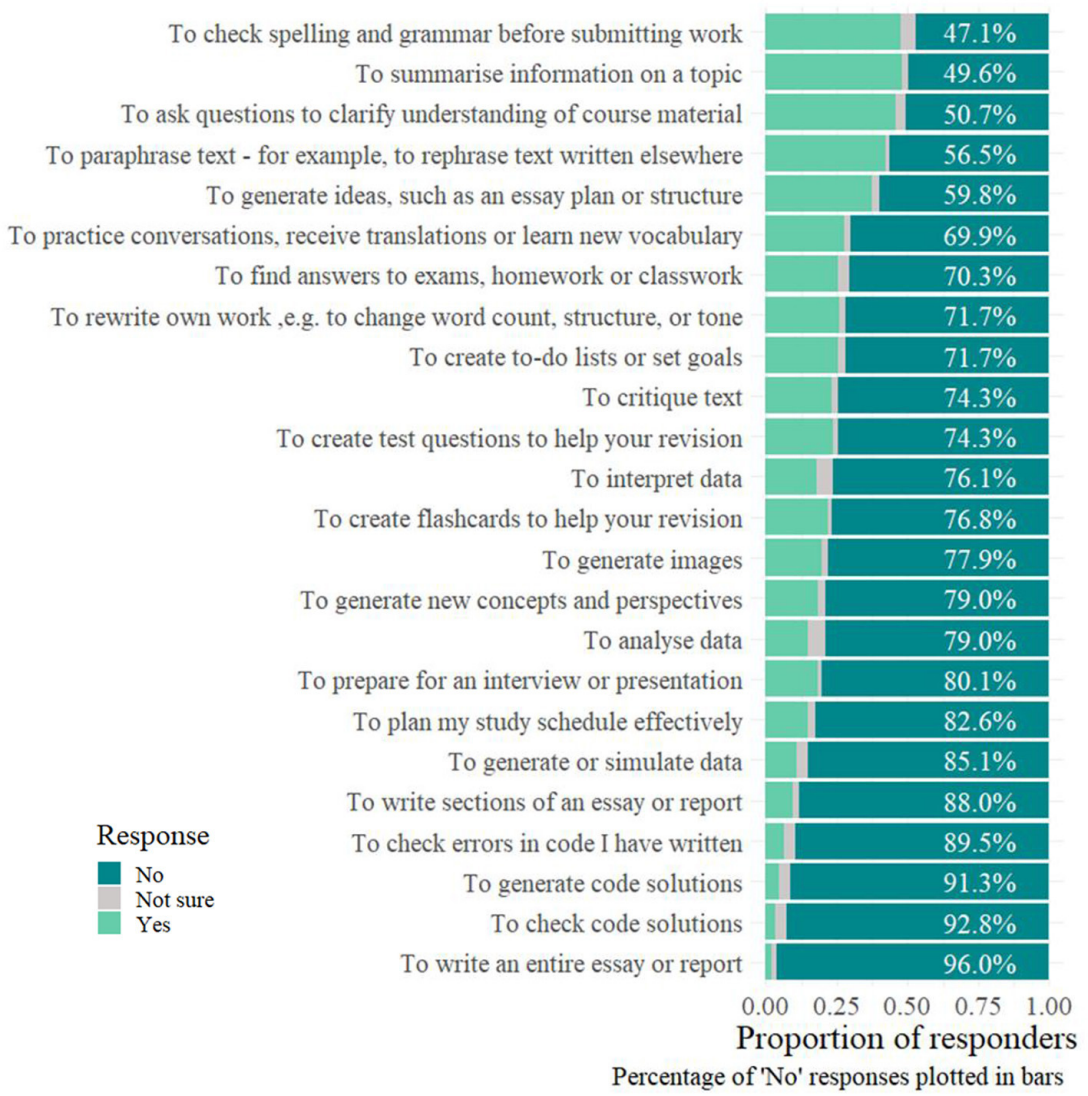
Proportion of participants responding to potential Generative AI uses.

When we specifically asked students gave for their studies, over half of them (*N* = 276, 60.9%) have already used AI tools to help with their degree. In free-text answers, the most common reason students gave for why they started using AI tools in their studies was because it “helps” (21%). We enumerated potential use cases and asked students to state whether they had used AI tools for these reasons (Figure 3).

For example, the most common reasons they gave for using AI from the options we provided were to summarize (*N* = 133, 48.2%) or paraphrase (*N* = 117, 42.4%) information, to run spelling and grammar checks before submitting work (*N* = 131, 47.5%), and to ask questions to help clarify their understanding (*N* = 127, 46.0%). In contrast, only 2.2% (*N* = 6) of students said they had used AI tools to write an entire essay or report. More details can be found in Figure 3.

The survey also included some open questions. Tables 5, 6 shows some quotes highlighting some of the common reasons students felt discouraged from using AI tools. The strongest reason was that they feared academic misconduct. However, students also were aware of AI's hallucinations, and were afraid it would reduce their learning.

**Table 5:** Common reasons students felt discouraged from using AI tools.

**Common themes**	**Example**
A fear of plagiarism and cheating	“It hasn't been written or produced by myself therefore I haven't earnt my work or rewards that I may receive by ‘cheating' with an AI tool.” “The fear of plagiarism”
The risk of inaccurate or outdated information	“That it can make stuff up which are false”
Reduced engagement and learning	“I feel more accomplished doing things by myself”
	“I don't want to depend on AI for my uni work. It'll be useless for me to bother attending uni then”
	“It wouldn't feel like I earned the grade”

**Table 6:** Secondary reasons students felt discouraged from using AI tools.

**Secondary themes**	**Example**
Mistrust	“I cannot trust AI […] Humans have emotions and morals, AI does not […]”
	“the way that they learn things about you.”
Regulatory constraints	“If there were rules against it.”
Ethical considerations	“I place a lot of value in human work and have ethical objections to the casual use of many AI tools that provide high speed imitations of human writing or artistic work, since they will likely outcompete the honest labor of the people whose work the models are originally built on […]”
Fear of the unknown	“Well, I am a bit scared of technology”

#### 3.1.3 What are students' impressions of the importance of AI to their future?

The next part of our survey covered students' impressions of the importance of AI to their future careers, or within their degree. We asked six questions (see Appendix A), including free-text boxes to explore the factors that might discourage students from using AI, questions they might want to ask their lecturers, and the future use-cases they identified. We also included Likert-type scales, which asked students to rate how likely they were to use AI in the future and the importance of understanding ethical AI use in their degree or future career.

Students have a range of topics that they would like to ask their lecturers about in relation to AI, but do not feel comfortable to do so. These include “using AI in your assignments,” “AI for paraphrasing,” “examples of AI,” and how to “use it properly.”

Overall, the majority of students (“Very likely” = 21.4%, *N* = 59; “Quite likely” = 41.3%, *N* = 114) felt that they would use AI tools in their degree in the future. When we asked if there was anything that would discourage them from using AI in their degree, the most common response was “plagiarism” (17%), with concerns also expressed around false (2%), inaccurate (1%), and incorrect information (1%).

**Figure 4:**
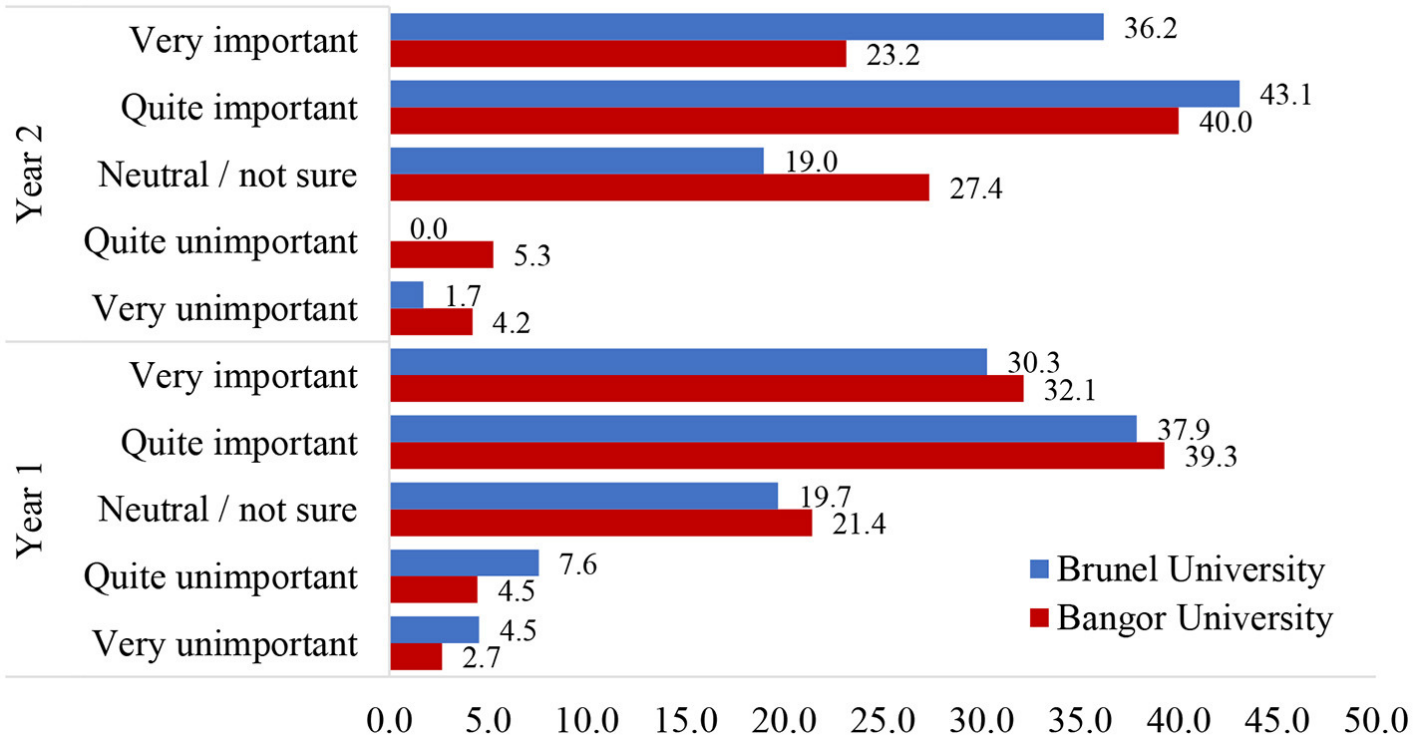
Percentage of responses to the question “*How important do you think knowing how to use AI ethically will be in your future career*?” NB, Only BSc years 1 and 2 from psychology were included due to the low numbers of students in the other categories.

**Figure 5:**
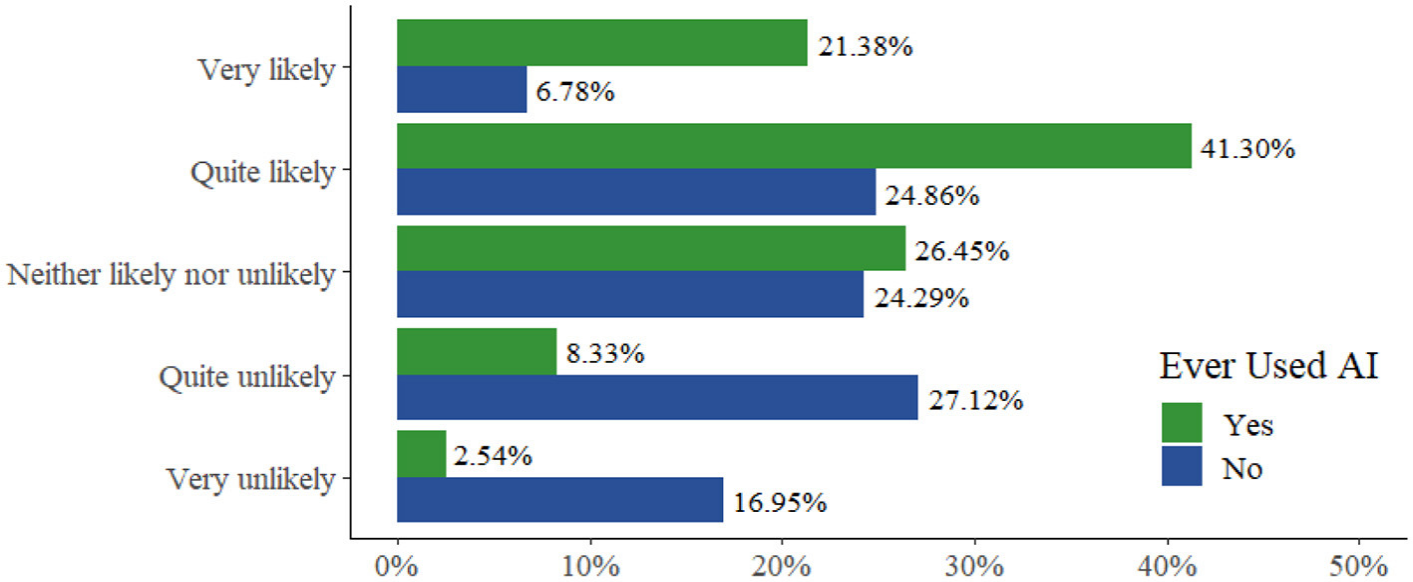
Percentage of responses to the question “How likely are you to continue to use AI (artificial intelligence) tools in your degree or future work?”.

Nearly two-thirds of students felt that the ethical use of AI would be important to them in their future careers (66.9%: “Very important” = 30.9%, *N* = 140; “Quite important” = 36.0%, *N* = 163). This aligns very closely with their view of the importance of being familiar with the ethics of AI during their current degrees (66.0%: “Very important” = 28.0%, *N* = 127; “Quite important” = 38.0%, *N* = 172; see Figure 4 for a breakdown by university and year for year 1 and 2 undergraduates in psychology). Overall, almost two-thirds of students felt that they would “continue to use AI tools” (62.7%: “Very likely” = 21.4%, *N* = 59; “Quite likely” = 41.3%. *N* = 114) in their degree or work in the future (see Figure 5 for a full breakdown).

Students who had used an AI tool were significantly more likely to believe that it is quite or very important to become familiar with the ethical use of AI tools during their degree (*M*_diff_ = 0.68; *t*_(322.95)_ = 6.72, *p* < 0.001, *d* = 0.67) and that it would be important to their future career (*M*_diff_ = 0.54; *t*_(313.63)_ = 5.12, *p* < 0.001, *d* = 0.52) compared to those who had never used an AI tool.

#### 3.1.4 University AI policies

**Figure 6:**
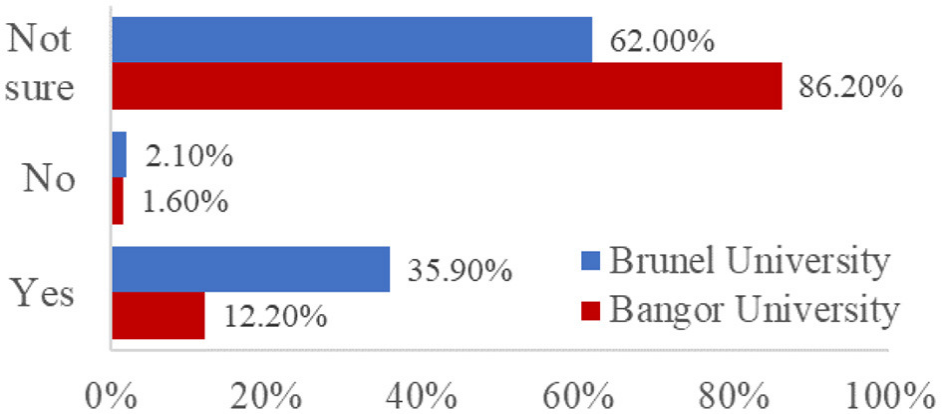
Percentage of responses to the question split by University “Does your university have a published Artificial Intelligence Use policy?”

**Figure 7:**
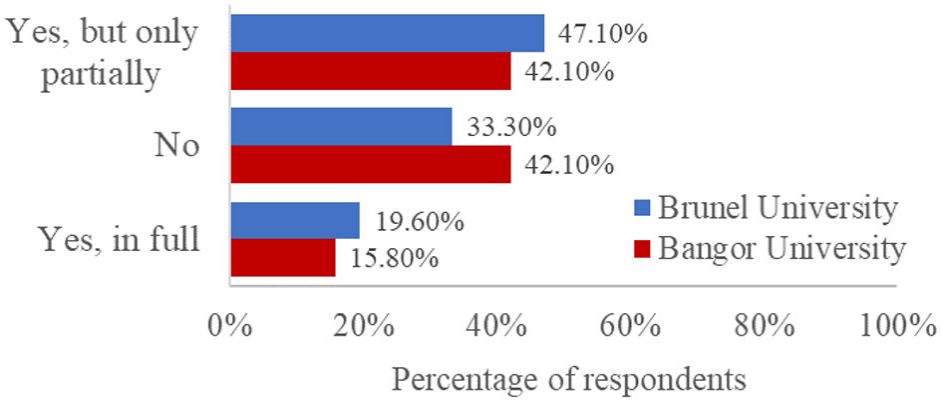
Percentage of responses to the question “Have you read it” to those who responded “Yes” to “Does your university have a published Artificial Intelligence Use policy?”

**Figure 8:**
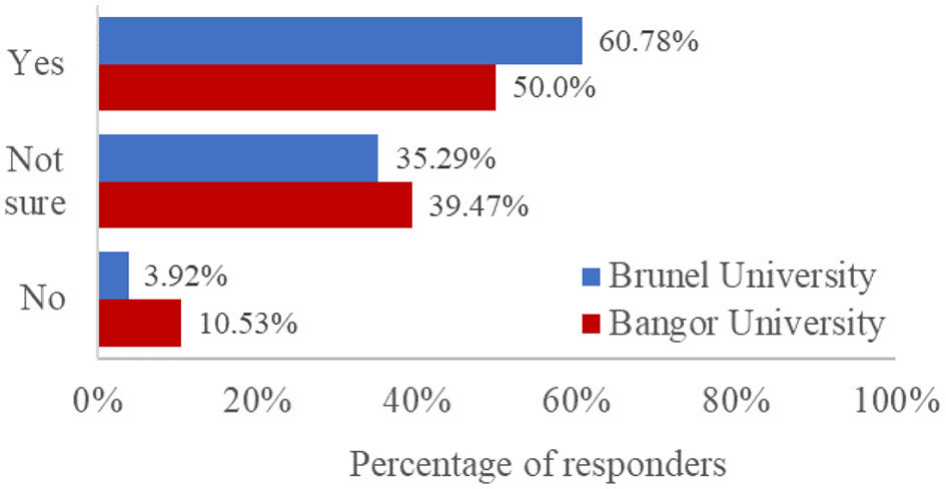
Of those who said “Yes, but only partially”, or “Yes, in full” to those who had read the university's AI policy, the proportion of responses to the question “Did you understand it?”

**Figure 9:**
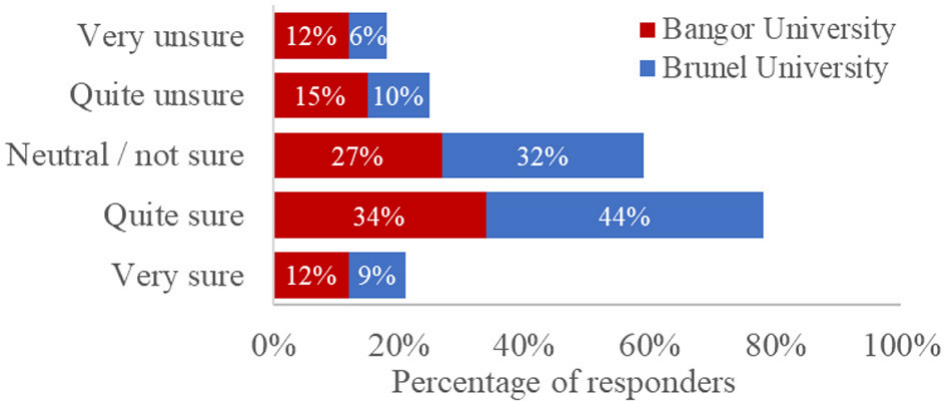
Percentage of responses, split by university, to the question: “How certain are you that you know what constitutes acceptable or unacceptable use of AI tools in your degree?”

Finally, our survey probed students' awareness and understanding of the AI policies published by their institutions. We asked four questions: three single-choice questions to determine whether they were aware of, had read, and understood AI policies, and a Likert-type scale to probe their level of understanding of the AI policies published by their institution (see Appendix A). As the AI policies published by Bangor University and Brunel University differed with regards to their length and readability, we present institution-specific data for comparison in Figures 6–9.

When we asked students whether their universities had published policies on the use of AI, which could reasonably be expected to help answer some of these questions, most participants were unsure (78.6%, *N* = 356; see breakdown by institution in Figure 6). Of those participants who were aware their university had published a policy (*N* = 89, 19.6%), only 18.0% (*N* = 16) had read it in full (Figure 7), and 56.2% (*N* = 50) felt they understood it (Figure 8). This suggests that students are not really aware of the AI policy. Our content design and readability analysis of both published AI policies (see Table 3) suggested that both could be much easier for readers to understand and follow, in terms of readability and cognitive load. Future consideration should also be given to the way in which policies are communicated to students; a standalone email may, for example, draw more attention to a new policy than including that information in a student newsletter that may not otherwise be read.

We asked all students if they felt they understood what constituted the acceptable use of AI in their degree (see Figure 9). Almost a quarter felt very unsure (10.2%, *N* = 46) or quite unsure (13.2%, *N* = 60). Most students were either neutral (28.7%, *N* = 130) or quite sure (37.1%, *N* = 168). Fewer students were very sure they understood acceptable use, as defined by their institution (10.8%, *N* = 49). Students who had never used an AI tool were significantly less confident that they knew what constitutes acceptable use than those who had [*t*_(451)_ = 3.05, *p* = 0.002, *d* = 0.29].

We ran an independent-samples *t*-test analysis to determine whether there were any differences between universities in students' awareness or understanding of AI policies, which may inform how institutions can most effectively communicate these policies with students. There was no significant difference in certainty around acceptable use (“*How certain are you that you know what constitutes an acceptable or unacceptable use of AI tools in your degree, ″t*(451) = −1.64, *p* = 0.102).

### 3.2 In-person focus groups themes

Through thematic analysis, 6 themes were identified. *Dependable support* consisted of the type of support students used AI for and how they may become dependable on it. The second theme of *Productivity* described how students used AI tools to boost productivity. *Creativity*, the third theme, describes students' perceptions on the relation between the use of AI tools and their own creativity. The fourth theme describes students' views on the *Ethics* of using AI. *Threat*, the fifth theme, explores students' views on the potential thread of AI for their future prospects. Finally, the last theme, *Human touch*, discusses the importance of including human interactions and not completely relying on AI.

#### 3.2.1 Theme 1: dependable support

The Dependable Support theme captures how students felt that AI was always available. Specifically, they felt AI was always there when they needed it, in both an educational and a personal capacity. Some noted this was not always a good thing as it could lead to overreliance and laziness. When students spoke about how they felt that AI was reliably there whenever they needed it, they discussed multiple contexts. For example, the immediacy with which it can help get things done “if you email a lecturer it might take like a day or two for them to respond but if you need like… an immediate answer to a… like an, an urgent question, AI can help you with that.” (P17, l. 112–114), and “it's more accessible. So, if you have a question about like an assignment and you don't understand, what it means, you can't always, access the lecturers” (P19, l. 107–109). Some students described AI like talking to a friend or social network, for example, “some people can use AI, to like, discuss their emotions” (P10, l. 537–538) and “it's like a really smart friend that you can have, that you can go to when you need it.” (P18, l. 118–119). There were even views that it could be a helpful tool for those with social anxiety: “It's almost like, cos in our generation I feel like there's a lot of like social anxiety that goes around and like… cos we have, got our phones that has everything like, we have so much trust in technology now, so the fact that we can then go to this like, AI and use it in an effective way for studies is like… so amazing.” (P18, l. 119–122).

However, some students also expressed concerns that the use of AI could lead to overdependence, “I feel like I was getting into this habit, like quite quickly of like, relying on it a lot” (P20, l. 285–286). Some even compared it to addiction “it can be very easy to get addicted to as soon as you start” (P18, l. 94). Some students suggested that this in turn can lead to negative consequences. This might be in an educational environment, “it can be, quite hindering to, someone's, learning” (P25, l. 751–752) or generally in society, “youuuu get into the habit and you get lazy” (P14, l. 89–90) and “Yeah, and if you, become, dependent on it it will hinder your, future works or, capabilities.” (P34, l. 354–355). Pragmatically, some students felt there was a balance to be achieved, “you shouldn't be, you shouldn't, hyper focus on, like rely on AI. You should use that as a steppingstone to (pause) your own work (clears throat) and, your own ideas” (P26, l. 355–357). This shows that some students were aware when and when not to use AI, and were still keen to develop their own skills.

Two main sub-themes arose around the theme of “Dependable Support”: firstly, the benefits of having a personalized assistant instantly available speaks to the themes of convenience identified in the survey. ChatGPT can effectively scaffold learners' efforts because of its within-session memory and ability to tailor content to students' specifications, similar to a skilled and responsive educator (e.g., Saye and Brush, 2002). Secondly, the theme of dependence or overreliance emerged frequently in the discussions. Whilst it has been shown that access to generative AI tools, such as conversational assistants, increases productivity (by 14% in Brynjolfsson et al., 2023), with the greatest impact on novice and low-skilled workers), there does not seem to be any research so far assessing the longer term cognitive consequences of using AI as a “thinking partner.”

#### 3.2.2 Theme 2: productivity

The Productivity theme is about how students perceive AI to enhance efficiency and output. As students, they noted that AI saves them time, and they expect this will also be true in their future careers. For example, students explained how they are already using AI to, for example, “summarize some of the, chapters in the book” (P14, l. 133), “use it to like kind of simplify, things” (P20, l. 125) and produce revision materials, “I know a friend that's, like, taken his notes, put it into, I don't remember what he used specifically, but got quiz out of it. So, used that to help his learning.” (P13, l. 175–176). They see it as “a faster way to access the information you, you want” (P34, l. 118). These quotes show how students are using AI tools to enrich their teaching and learning.

In addition, students feel that AI is going to be helpful for enhancing skills, particularly writing. For example, “Sometimes some erm, some people, can't write professionally, ((laughs)) so some-sometimes they're using, AI tools to help them write professional emails.” (P24, l. 156–157). They also feel that it is helpful where there are repetitive tasks or processes that do not require too much active thought, such as transcription, “… but I have found that, it's, helped, me, in transcribing quite a bit….” (P17, l. 175–178). A further advantage noted was AI's ability to deal with admin: “if you've done all of the administrative things with AI, then you have more time to deal with like more complex problems.” (P16, l. 638–639) and “having more free time to think about like, more complex stuff.” (P16, l. 643–644). And “it makes, life, slightly, easier” (P26, l. 243). Overall, students see the use of AI in these contexts as positive.

In conclusion, students used AI tools in a variety of ways to boost productivity. Like Brynjolfsson et al. (2023), who saw that AI use prompted greater performance improvements in novice and low-skilled workers, the use of ChatGPT has been shown to substantially raise productivity in writing tasks undertaken by college-educated professionals (Noy and Zhang, 2023). Noy and Zhang were able to reduce inequality in productivity by generating a greater improvement in writing tasks in those with weaker skills, decreasing average task time by 40% and increasing output quality by 18%. This suggests that AI tools might be crucial in reducing educational inequalities. Personalizing AI training, rather than issuing general guidance to students, would facilitate a more nuanced and individual assessment of the potential productivity gains that could be made using generative AI.

#### 3.2.3 Theme 3: creativity

The Creativity theme captures students' views on AI supporting the generation of ideas and content. Students discussed the creative use of AI in multiple contexts, including to “Generate different measures or scales” (P11, l. 69), “just to get you like a starting point for the essay” (P17, l. 103–104). Many students mentioned how they found this helpful to them, “think it can enhance your, creativity” (P16, l. 636). However, some students disagreed, for example, “Long periods of time with no issue, but some people may struggle with that. So they can, AI can actually help them, whereas with me, it can actually make me less creative, make me worse.” (P17, l. 660–662). They felt that AI can actually “reduce your creativity” (P25, l. 750) and leads to you not being “as creative as you usually would be” (P6, l. 123). Thus there seem to be opposing views regarding whether AI boosts or stifles creativity.

Concerns around negatively affecting one's own creativity have been explored in the literature since the generative AI boom; in fact, a new psychological term named “Creative Displacement Anxiety” (Caporusso, 2023) has recently been described as a psychological state caused by the perceived or actual threat of the replacement of human creativity by advanced generative AI technologies. Caporusso also described possible mitigation strategies and interventions to assist in the use of AI as a creative tool, such as comprehensive education to demystify AI and establishing ethical guidelines and policy measures to ensure responsible AI integration. O'Toole and Horvát (2024) recently proposed using Generative AI models to understand the creative process, and develop interfaces to encourage creativity and ideation in the creative process.

#### 3.2.4 Theme 4: ethics

The Ethics theme addresses students' concerns as to the appropriate use of AI; how much use is fair without cheating. It also captures the ethical considerations around access to AI, and what it does with the data you give it. When discussing the use of AI in their studies, students expressed concerns around whether AI should be used at all, “I don't think that… AI has any… use in like that sort of education or should have any use in that sort of education.” (P29, l. 61–62), and “in my opinion at this point you should kind of already have a g-good idea of what is put into an essay.” (P29, l. 64–65). They also expressed views that use of AI on any level was “cheating” (P4, l. 83) and that those students had “cheated their way through” (P4, l. 497) their degrees. Where students were happy with the idea of using AI to support their studies, some expressed concerns that they were unsure whether “we're allowed to do or how we would” (P10, l. 46), and whether they “would be at risk of like plagiarism” (P10, l. 198) and “false flags of plagiarism” (P29, l. 295–296).

They were also concerned about the ethics of the information Generative AI provides and infringing on intellectual property rights of others, “where we talk about like, IP and like people's actual words, I think AI kind of blurs the line of… what… is someone's work… and how it gets abused” (P27, l. 254–257). In addition students raised concerns about the biases around AI use, “[I] know it can be, very biased,” (P17, l. 453–454), and “could be, possibly like, racist” (P23, l. 406–407), and its trustworthiness, “it isn't kind of trustworthy” (P9, l. 350–351). These concerns around IP of information, trustworthiness and bias extended as far as the ethics of how the information of vulnerable users may be used without their knowledge or understanding of the implications, “introducing people who, may not be familiar with AI, may not even be, that erm, developed mentally, er to AI” (P12, l. 564–565). Apart from the positive aspects of AI, these quotes show that students were concerned about the ethics of AI use.

The two principal subthemes within the “ethics” theme were internal, relating to a fear of plagiarism or a lack of ownership of the work they produced, or external, concerning the impact of AI on others, such as the plagiarism of creators' work or the potential to propagate bias or inaccurate information. Plagiarism is not a new concern for students and, in fact, has been the focus of much research in recent years. A study by Gullifer and Tyson (2010) investigating university students' perceptions of plagiarism revealed a complex understanding of the issue. Students often struggled to differentiate between acceptable citation and plagiarism, and the fear of unintended plagiarism and harsh penalties was also prevalent due to a perceived lack of clarity around the topic. These strongly echo the concerns students feel around AI use and highlight the need to extend the work educators do with students on academic integrity to explicitly include the ethical use of AI.

#### 3.2.5 Theme 5: threat

The Threat theme reflects the perceived threats that students believe AI poses, particularly in terms of threats to jobs. It also considers how those threats might be mitigated. Students expressed views that AI is going to be widespread, “it is just a matter of time before erm, AI does get… introduced to our everyday life” (P11, l. 250–251) and “it's gonna take over the world” (P1, l. 96). Some students felt that AI already does or eventually will pose a threat to work, “it's already putting people out of jobs in some, aspect” (P11, l. 271–272), “our jobs, will definitely be threatened” (P7, l. 407–408). One student even felt that rather than AI working for us, it would be the reverse, “does that kind of sound like we work for AI in the future?” (P331, l. 474). However, other students felt it would be a more balanced future, “AI in jobs, would be helpful for a push in the right direction” (P8, 1. 452–453), “not necessarily, threatening our entire jobs but maybe… making us perform them more efficiently” (P9, 1. 443–445). Having discussed the positives of AI, students also noted how AI is going to change the workplace and how it may affect their future.

Students' views on the importance of AI to their future centered on work and the threat AI poses to their jobs and the economy. Despite this, several participants took a balanced view and believed that AI would become a partner in the workplace, increasing efficiency. No participants felt that AI would become unimportant. These views echo research studies that suggest AI can improve productivity, efficiency, and job performance (Makridakis, 2017) while freeing up employees for more strategic tasks (Frank et al., 2019), and other studies that highlight potential job displacement and increased inequality (Wang and Siau, 2019). These concerns highlight the need for more AI skills to be embedded in education so that students can be empowered to use the right tools for the right tasks in their future careers.

#### 3.2.6 Theme 6: human touch

The final theme, Human Touch, considers how increased interaction with AI might affect our personal interactions. Students discussed instances where AI is better than humans, “A student asks, let's say a teacher a question who is, I don't know, like a AI, something, they'll be able to give you whatever they want cos they have the plethora of the internet behind them. Whereas, humans only have a limited amount of, knowledge.” (P11, l. 273–276). However, they felt that a human touch was still beneficial in some roles, such as for therapists, “use of AI in, maybe like, therapy, and stuff won't be… that good of a use cos at the end of the day a patient… will want a erm, sort of emotional connection, with… the therapist.” (P10, l. 317–319), or where there are “non-verbal cues that an AI won't be able to detect” (P13, l. 371). They also felt that in some instances, it was important for a human to be present to encourage certain behaviors, “you could be told by a person, this is what you have. You'd be more likely to ((pause)) challenge it, or discuss it further with a human than with a robot kind of thing.” (P11, l. 326–328). There was also a strong sense that use of AI reduced a person's personality and lost the human touch in their interactions. For example, “your emails are gonna come off as like, eventually as cold. You're not gonna be, the, like… the personal input that you'll have while writing the email, like the personal touch,” (P17, l. 650–652). This final theme discussed the importance of keeping a human touch, which students felt may not always be possible with AI.

Like our participants, studies have noted differences between human-to-human and human-to-AI interactions. Studies released before ChatGPT showed that when engaging with AI, users typically demonstrated reduced levels of openness, agreeableness, extroversion, and conscientiousness compared to interactions with human friends (Mou and Xu, 2017), and may provoke stronger reactance and a diminished sense of autonomy among users (Sankaran et al., 2021). Individuals who form friendships with AI, such as through more modern social chatbots, perceive and interpret these relationships differently from human friendships (Brandtzaeg et al., 2022). It remains to be seen whether advances in large language models are able to more closely mimic human-to-human interactions and how such advances will be received by humans.

## 4 Discussion

This study probed students' familiarity with AI tools, their views on its use cases, their impressions of its importance to their degree and in their future careers, and their subjectively perceived understanding of institutional AI use policies.

### 4.1 How do students at all levels of study currently perceive and use AI tools?

The levels of familiarity varied; although the vast majority of students had heard of AI tools, two thirds had used them and the remaining third had not. This highlights the need to educate students on the possible use cases for AI tools to avoid a new digital divide from emerging, particularly given the Quality Assurance Association's statement on the inclusion of AI skills as a new graduate attribute (Quality Assurance Agency, 2023).

Over half of the students in this study had used AI tools to help them with their degree, often motivated by curiosity, a desire for greater productivity, or better quality work. This finding underscores a need to conduct further research to understand the ways in which the effective use of AI tools may benefit students. This accords with recent studies that have explored higher education students' perceptions of AI use in their degrees, which have revealed a generally positive attitude toward AI in teaching and learning, and its potential for personalized support, writing assistance, and research capabilities (Chan and Hu, 2023).

Saving time and increasing productivity emerged as strong themes in our study, which accords with the findings of Lai et al. (2024), who explored the relationships between intrinsic motivation, perceived usefulness, and the perceived ease of use of ChatGPT on students' intentions to use the technology. Although Lai et al. (2024) did not find that perceived ease of use significantly influenced behavioral intention, they did find that intrinsic motivation and perceived usefulness were strong predictors of students' intentions to use ChatGPT. Surprisingly, the perceived ease of use may be less critical for AI technology adoption among digitally comfortable users. Understanding whether AI use affects students' educational attainment will be critical to evaluating how the technology may be used in education in the future.

This is particularly pertinent given recent evidence regarding how AI-generated content is subjectively evaluated by humans. Proksch et al. (2024) investigated whether texts, purportedly written by either a human or ChatGPT, were perceived by human readers. Though all experimental texts were written by ChatGPT, the authors found that ChatGPT was consistently rated lower than allegedly-human authors. Students using AI tools to help with their writing, in a way that is sanctioned by their assignment guidelines, must be made aware of these potential drawbacks of perceived AI use. Moreover, educators must also recognize their own biases (e.g., Luo, 2024; Proksch et al., 2024), which may lead them to unfairly penalize students legitimately using AI tools, for example by downgrading them or invoking academic dishonesty procedures—a concern raised by students in this survey, who were afraid of the *possibility* of plagiarism occurring if they used AI tools (see Table 4; also see Luo, 2024), suggesting they did not feel in control of whether or not plagiarism took place.

Consistent with the implications of negative perceptions of AI use in academic settings, a significant barrier to interacting with AI tools, which students expressed in the survey, was their fear of plagiarism and cheating. This view was shared by students in a focus group setting and by 51% of students in a separate survey of 1,000 students (BestColleges.com). This apprehension extends beyond the concern of plagiarism to a broader fear that any use of AI might lead to accusations of academic dishonesty, exacerbating students' reluctance to engage with these technologies. Whilst universities' published AI policies seek to delineate acceptable use, it is clear that the policies must be communicated more effectively to ensure students are conversant with the guidelines. A recent study by the Higher Education Policy Institute (HEPI, 2024) showed that 12% of 1,200 students surveyed felt their institution's AI policy was not clear. Similarly, in the present study, fewer than 20% of students surveyed knew their institution had an AI policy, and half of those who had read the policy felt it was clear.

### 4.2 What are students' impressions of the importance of AI to their future careers?

Students were both optimistic and cautious about the potential for AI to enhance their academic and professional lives, but expressed concerns about its ethical use. The majority of students expressed a likelihood of using AI tools in their degrees, despite concerns about plagiarism and the accuracy and reliability of information provided by AI. Nearly two-thirds of students believed that ethical use is critical, highlighting the importance of integrating ethical training into AI education, ensuring that students are not only proficient in using these tools but also aware of the broader implications of their use.

Students' views on AI's future impact on work were mixed. While some students feared job displacement, a reduction in human creativity, and economic threats, others saw AI as a partner that can enhance efficiency and productivity. These perspectives echo broader research findings that highlight both the potential benefits and risks of AI in the workplace (Makridakis, 2017; Frank et al., 2019; Wang and Siau, 2019), and align with research suggesting that AI can enhance productivity. Studies by Brynjolfsson et al. (2023) and Noy and Zhang (2023) have shown that AI tools can significantly improve performance in writing tasks, suggesting potential for reducing educational inequalities. Future work should explore how employers view the need for AI skills to be developed among prospective graduate hires, and how universities can support this new graduate employability attribute (Quality Assurance Agency, 2023).

### 4.3 What awareness do students have of AI policies within their universities?

We asked students if they were aware of their institution's AI policies and whether they understood what constituted the acceptable use of AI in their degree. The responses varied widely, with most being unaware of AI policies, and not all those who were aware of them had read them, and not all who read them understood them. There were no differences in students' understanding of AI policies across the two institutions. This suggests that current communication strategies are insufficient and that policies need to be more accessible and comprehensible.

Readability is essential for effective communication and encompasses various technical elements such as sentence structure, vocabulary, and word length, as well as qualitative aspects like legibility and content layout (Klare, 1963). The study of readability emphasizes that comprehension is more about the reader's experience than the author's intent, factoring in audience-specific elements such as reader competence and motivation (Klare, 2000).

Research consistently shows that texts deemed easy to read by the intended audience enhance comprehension and retention (Carver, 1992). We examined the AI policies of Brunel University London and Bangor University from a content design and readability perspective. Brunel University's policy, with a Flesch-Kincaid Grade Level of 11.8, is more challenging to read compared to Bangor University's level of 9.6. Despite being easier in terms of grade level, Bangor's policy had a lower Reading Ease Score of 38.8, indicating that it is still quite difficult for readers. The complexity is further illustrated by the average words per sentence and syllables per word, where Brunel's sentences are longer but less syllable-dense than Bangor's.

Bangor University's policy was substantially longer than Brunel's more concise document. The length of Bangor's policy may contribute to cognitive overload for students, making it harder to digest and understand the content. Brunel's shorter, more succinct policy might be easier to manage but still presents challenges in readability and would benefit from breaking down long sentences. Both policies would benefit from simplified language and a clearer structure to help students navigate and understand the content better. These changes would lower the Flesch-Kincaid Grade Level and thereby improve comprehension in students (Carver, 1992).

The lack of awareness and understanding of AI policies are likely to have contributed to the ethical concerns students expressed about AI. Students worried about both personal academic integrity and the broader implications of AI, such as propagating bias or inaccurate information. These concerns echo findings from previous research on plagiarism (Gullifer and Tyson, 2010), highlighting students' struggles to differentiate between acceptable citation and plagiarism and their fear of unintended plagiarism due to unclear guidelines, and other studies that have shown that whilst students are supportive of tools like Grammarly, the majority are unsupportive of using ChatGPT for entire essays (Johnston et al., 2024).

In addition to clarifying local AI policies on acceptable use, there is a need to educate students on the effective use of generative AI tools. As some students noted, relying too heavily on AI may reduce the sense of accomplishment and self-efficacy they gain from completing their degree. Rather than using AI to generate work, students must be educated on the ways they can use AI as a partner, allowing them to retain their creative control over their work and support their academic development. Recent studies have attempted to quantify some of the cognitive effects of using Generative AI as a study partner. For example, Essel et al. (2024) employed ChatGPT as an academic intervention in an undergraduate research methods course and found that students who were asked to use ChatGPT, vs. a control group who were asked to use traditional databases and search engines, exhibited higher levels of critical thinking, reflective skepticism, and critical openness on a self-report scale. Longer-scale interventions and more objective measures would strengthen these promising early findings.

### 4.4 Recommendations

Recommendations emerged from the findings of this study. As there is quite a disparity in students' awareness of AI tools and how to use them, programmes might consider including dedicated teaching sessions to facilitate this. These should be done at subject-level as each subject may use different AI tools in the field. During these sessions, students can also be made aware of the university's AI policy and guidance. The authors from Brunel University London have implemented these sessions in their programme at levels 4–6 which were positively received by students. Secondly, AI plays a role in assessments too as we have seen through how some students use AI. Academics have a variety of options here: Keep their assessment as is even if it is not completely AI-resistant; move to in-person exams, however these often do not support students in the development of transferable skills and rely heavily upon recall; embed AI into the assessment whereby students need to use AI tools in their work and then either reflect on it or critique the AI tools' output. At Brunel University London, the latter approach was implemented in two assessments. In one, students were previously required to write a written reflection. These were typically very descriptive, with students unable to provide a critique. This year when the students also had to critique a ChatGPT output, there was a noticeable increase in their critical thinking abilities. In a second adapted assessment, a traditional essay was altered to require students to incorporate a critique of a ChatGPT output in their work. Critical thinking ability was again substantially greater than previous years. This feedback was received independently from two different module leads.

### 4.5 Limitations

This study has several limitations. Firstly, the demographics of the participants, including gender, ethnicity, socioeconomic status, and access to technology, were not captured in the survey. These variables could have provided a more comprehensive understanding of the findings as they may influence students' access to, and familiarity with, AI tools. The study's UK context, along with its focus predominantly on psychology students, limits the generalizability of the results to other disciplines and regions.

Despite the survey being anonymous, social desirability bias may have influenced what students were willing to disclose about their AI use. Psychology and sport science students might conceivably realize that using AI to write an entire essay would be considered unethical, while using it to write code might be acceptable in the context of UK undergraduate psychology studies. However, most students at Bangor and Brunel universities use SPSS and are not required to code as part of their degree, further affecting whether they are likely to disclose such use cases as part of this survey. Therefore, how students are currently using AI in their degree should be viewed with caution regarding the context of these students.

Given the rapid evolution of AI technology, these findings on the perceptions of AI may quickly become outdated. However, the importance of understanding students' fears and uncertainties regarding AI are likely to persist even as the tools themselves change. This is similar to ongoing concerns about plagiarism, where the fundamental issues of acceptable use remain despite advancements in technology.

## 5 Conclusion

The present study revealed a mixed response regarding students' familiarity with AI tools and their opinions on appropriate uses. Among those aware of AI tools, ChatGPT was predominantly used; however, a significant portion had yet to engage with these technologies, underscoring the necessity for targeted education to bridge this gap. Most students emphasized the importance of understanding AI tools for their current studies and future careers, and there was a clear desire among students to learn more about AI and its applications. Students who did use AI tools often did so for improved productivity, however, concerns about plagiarism and ethical use posed barriers to adoption. Findings from focus groups aligned closely with the survey data, highlighting students' strong interest in receiving dedicated support on using these tools. There were mixed feelings toward AI's future impact on careers, but students recognized that future employers may value these skills. Consequently, academic programmes should consider incorporating sessions to develop key AI competencies. Additionally, the improved understanding and communication of AI policies will not only alleviate students' fears but also prepare them for responsible and effective use of AI in their academic and professional lives. This research supports a long-term goal to enhance teaching, learning, and student engagement through AI and effective technology use.

## Data Availability

The datasets presented in this study can be found in online repositories. The names of the repository/repositories and accession number(s) can be found below: https://pure.bangor.ac.uk/admin/workspace.xhtml.
