# Supplementary material for: The impact of AI on education and careers: What do students think?
Source: Front Artif Intell. 2024 Nov 14;7:1457299. doi: 10.3389/frai.2024.1457299 (PMC11602497; doi:10.3389/frai.2024.1457299)
Supplement: Supplementary file 1 [file Data_Sheet_1.docx]

**Appendices**

**Conflict of Interest**

The authors declare that the research was conducted in the absence of any commercial or financial relationships that could be construed as a potential conflict of interest.

**Author Contributions**

All authors: Conceptualization, Data curation, Formal analysis, Investigation, Methodology, Project administration, Resources, Visualization, Validation, Writing – original draft, Writing – review & editing.

**Funding**

No funding was received for this study.

**Acknowledgments**

N/A

**Data Availability Statement**

The datasets analysed for this study can be found in the PURE REPOSITORY. [https://pure.bangor.ac.uk/admin/workspace.xhtml].

**Appendix A Survey Questions**

| **Number** | **Type** | **Question or statement** | **Format** |
| --- | --- | --- | --- |
| 1 | Screening | Have you read the information sheet? | Yes / No |
| 2 | Screening | I am over the age of 18. | Yes / No |
| 3 | Screening | I understand that no personal identifying data is collected in this study, therefore I know that once I have submitted my answers, I am unable to withdraw my data from the study; the personal data collected in the study is only used to determine the winners of the raffle and will be removed once the raffle is concluded. | Yes / No |
| 4 | Screening | I agree that my data can be anonymised, stored and used in future research in line with Bangor University and Brunel University’s data retention policies. | Yes / No |
| 5 | Screening | I agree to take part in this study. | Yes / No |
| 6 | Admin | What is your email address? We will only use this to contact you if you win a prize or to give you course credits. | Free text |
| 7 | Admin | What is your phone number? We will only use this to contact you if you win a prize. | Free text |
| 8 | Demographic | Which university are you studying with? | Free text |
| 9 | Demographic | What degree programme are you following? | Free text |
| 10 | Demographic | What is your current level of study? | Single choice question: BA; BSc; MA; MSc; MSci; Professional doctorate; PhD; Other |
| 11 | Demographic | What is your current year of study? | Single choice question: Year 0 / Foundation year; Year 1; Year 2; Year 3; Year 4+ |
| 12 | AI: perception | Have you heard of AI (artificial intelligence) tools? | Single choice question: Yes; No; Not sure |
| 13 | AI: perception | List the AI (artificial intelligence) tools you have heard of. If you have not heard of any, enter ‘none’. | Free text |
| 14 | AI: perception | Have you ever used an AI (artificial intelligence) tool? | Single choice question: Yes; No; Not sure |
| 15 | AI: perception | Which AI (artificial intelligence) tools have you used? | Free text |
| 16 | AI: perception | How confident are you with the AI (artificial intelligence) tools you’ve used, in general? | Likert scale: Very confident; Quite confident; Neutral/not sure; Not very confident; Not confident at all |
| 17 | AI: perception | Could you please explain your confidence rating | Free text |
| 18 | AI: perception | How have you used AI (artificial intelligence) tools within your degree? | Single choice question: Yes; No; Not sure for each of the following:  To run checks, such as spelling and grammar, in a piece of work before submitting it; To generate ideas, such as an essay plan or structure; To write an entire essay or report; To write sections of an essay or report; To rewrite work you have written, for example to reduce the word count, change the structure or its tone; To ask questions to clarify your understanding of lecture, seminar or course material; To find answers to questions you've been given for exams, homework or classwork; To create test questions to help your revision; To create flashcards to help your revision; To summarise information on a topic; To generate images; To generate or simulate data; To analyse data; To interpret data; To generate code solutions; To check code solutions; To check errors in code I have written; To generate new concepts and perspectives; To practice conversations, receive translations or learn new vocabulary; To create to-do lists or set goals |
| 19 | AI: perception | How have you used  AI tools within your degree? | Single choice question: Yes; No; Not sure for each of the following:  To plan my study schedule effectively; To paraphrase text - for example, to rephrase text written elsewhere; To prepare for an interview or presentation; To critique text - for example, to highlight weaknesses in a study design, identify conclusions that are not supported by a study, or to find spelling or grammar errors. |
| 20 | AI: perception | Why did you decide to try the AI tools you have used? If you've used more than one, please give a reason for each. | Free text |
| 21 | AI: perception | Are there any other ways you think you might use AI (artificial intelligence) tools in your degree or work in the future? | Free text |
| 22 | AI: future | Future AI (artificial intelligence) tool use | Likert scale: Very likely; Quite likely; Neutral/not sure; Quite unlikely; Very unlikely |
| 23 | AI: future | How likely are you to use AI (artificial intelligence) tools in your degree in the future? | Likert scale: Very likely; Somewhat likely; Neutral/not sure; Somewhat unlikely; Very unlikely |
| 24 | AI: future | How do you think you might use AI (artificial intelligence) tools in your degree or work in the future? | Free text |
| 25 | AI: future | What would discourage you from using AI (artificial intelligence) tools in your degree? | Free text |
| 26 | AI: policies | Does your university have a published AI (artificial intelligence) policy? | Single choice question: Yes; No; Not sure |
| 27 | AI: policies | Have you read it? | Single choice question: Yes, in full; Yes, but only partially; No |
| 28 | AI: policies | Did you understand it? | Single choice question: Yes; No; Not sure |
| 29 | AI: policies | How certain are you that you know what constitutes acceptable or unacceptable use of AI (artificial intelligence) tools in your degree? | Likert scale: Very sure; Quite sure; Neutral/not sure; Quite unsure; Very unsure |
| 30 | AI: future | How important will the ethical use of AI (artificial intelligence tools) be to you in the future? | Likert scale: Very important; Quite important; Neutral/not sure; Quite unimportant; Very unimportant, for each of the following:  How important is it for you to become familiar with the ethical use of AI tools during your degree?; How important do you think knowing how to use AI (artificial intelligence) tools ethically will be in your future career? |
| 31 | AI: future | Is there anything you wish you could ask or say to your lecturers about AI use, but don't feel comfortable asking them directly? | Free text |
| 32 | AI | Are there any final comments would would like to add? | Free text |
| 33 | Admin | May we contact you at a later date to update your answers or complete a new survey about AI use? | Single choice question: Yes; No |

**Appendix B Focus Group Schedule**

1. What do you think AI means?
2. How do you think AI can be useful in education?
3. Is AI already part of your programme?
   1. How?
   2. Where?
   3. What does it involve?
4. In your view how can AI be used in your programme?
   1. Examples, details
5. Why do you think it is important to include using AI in education?
6. Which AI skills do you think are useful to learn?
7. Are there any problems with using AI in education?
   1. If so what?
   2. Why?
8. Which AI tools do you know?
   1. How are you using them?
